# The ‘jimble’, a southern Australia box jellyfish, *Carybdea rastonii* Haacke, 1886: clinical symptoms, first-aid treatments and species distribution

**DOI:** 10.1016/j.toxcx.2026.100254

**Published:** 2026-04-01

**Authors:** N.E. Meyler, M.L. Mitchell

**Affiliations:** a Toxinology Department, Women's and Children's Health Network, North Adelaide, South Australia, 5006, Australia; b School of Medicine, College of Health, Adelaide University, Adelaide, South Australia, 5005, Australia

**Keywords:** Marine envenomation, Cnidaria, Toxinology, Public health, Epidemiology, Dive hazards

## Abstract

Within the field of jellyfish first-aid treatments, we have identified a distinct lack of evidence-based clinical research, which is most notable among species considered non-lethal. One such species is the southern Australia cubomedusa, *Carybdea rastonii* Haacke, 1886, affectionately known as the ‘jimble’. Little is understood about the symptoms of envenomation or the distribution of this species throughout its Australian range. Evidence infers that there is no effective first-aid treatment to alleviate pain from its sting, which may last up to 24 h. Variant spellings of the scientific species name and local common names complicate our understanding of first-aid research. Species misidentification and errant distributions can confound appropriate first-aid treatment. Here, we synthesise evidence of clinical symptoms, past and current first-aid treatments related to *Carybdea rastonii* envenoming. In addition, we provide a brief taxonomic overview of *Carybdea* Péron and Lesueur, 1809*,* highlighting the frequent misidentification of *Carybdea* species and define future research streams.

## Highlights

• There are no evidence-based first-aid studies for *Carybdea* species.
• Current treatment protocols do not effectively relieve pain associated with *Carybdea rastonii* envenomation.
• Hot water immersion treatment (e.g. showers) may not be suitable for SCUBA divers due to the risk of developing decompression sickness.
• *Carybdea rastonii* is commonly misidentified in studies and museum collections.
• There is a clear need for revision of *Carybdea* species occurring in Australia correlating to geographical distribution.

## Graphical abstract

**Figure:**
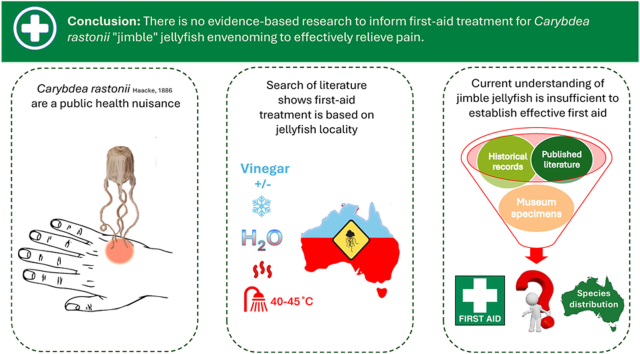

## 1 Introduction

The Phylum Cnidaria is one of the oldest venomous animal lineages, preceding the Cambrian period (Park et al., 2012). They are a fauna commonly encountered by humans in the marine environment (Remigante et al., 2018). Jellyfish (Scyphozoa Goette, 1887 and Cubozoa Werner, 1973) represent a significant percentage of species within the phylum. Clinical symptoms following jellyfish envenomation events can vary widely in severity, depending on the species responsible (Kamsani et al., 2026; Peng et al., 2024; Williamson et al., 1996).

Box jellyfish (cubomedusae) stings produce a wide range of symptoms. There are currently 51 recognised species of box jellyfish (Collins, 2025). Stings from many jellyfish species do not result in permanent damage to the afflicted area or person (Ballesteros et al., 2021), although they have the potential to produce fatal reactions. The well-known *Chironex fleckeri* Southcott, 1956, the Australian box jellyfish, has caused multiple fatalities. Since 1980, in one Australian state there have been 10 deaths, with less than five deaths between 2006 and 2022. Other box jellyfish, including some carybdeids, cause the potentially fatal Irukandji syndrome, which may occur in severe envenomation events (Fenner and Hadok, 2002; Gershwin, 2007; Gershwin et al., 2013). Irukandji syndrome is associated with the species *Carukia barnesi* Southcott, 1967, *Malo kingi* Gershwin, 2007, and *Carybdea xaymacana* Conant, 1897 (Tibballs et al., 2012).

Many studies on jellyfish envenoming focus on species which are the most adverse to public health, like *Chironex fleckeri,* i.e., have the potential to be lethal (Cegolon et al., 2013; Gershwin, 2007). Species which can cause harm, but are not thought to cause permanent damage, receive little attention. One such species is *Carybdea rastonii*, commonly known as the “jimble”, a small box jellyfish common to southern Australia (Tibballs, 2006). *Carybdea rastonii* is highlighted as a marine stinger of note for recreational beachgoers (Fenner and Williamson, 1987; pers. comms. J. Baker, 2024; Surf Life Saving Australia, 2020).

*Carybdea rastonii* is most commonly seen in autumn (State Library of South Australia (SLSA): PRG 233/9.10, 1958) to winter (Gershwin, 2023), is found in shallow coastal waters to 30 m depths (Cleland and Southcott, 1965; Rowland and Eipper, 2018), and in the open ocean (SLSA: PRG 233/9.10, 1959). *Carybdea rastonii* is characterised by its box-shaped bell with white “stinging warts” on the surface and four, single tentacles (Haacke, 1886), which are pinkish in colour (Acevedo et al., 2019).

Cubozoa are often highly regional (Gershwin, 2005). *Carybdea rastonii* was previously thought to be widespread throughout the Pacific, including southern Californian, Japanese and Australian waters. (Acevedo et al., 2019). Current treatments for jellyfish are recommended based on geographical location (ANZCOR Australian and New Zealand Committee on Resuscitation, 2025). Jellyfish first-aid treatments may have varying efficacy due to differences between species (Cegolon et al., 2013), geographical region and life stage of the jellyfish (Seymour, 2017).

There is inadequate knowledge of appropriate, evidence-based first-aid treatment for many species of jellyfish (Cegolon et al., 2013). We undertake a search of the available literature (published and unpublished) on *Carybdea rastonii*, to highlight research gaps that impact current interpretations of first-aid best practice. We collate clinical symptoms, evidence-based first-aid studies, taxonomic nomenclature, taxonomic identifications in literature and museum collections, and species distribution.

## 2 Materials and methods

### 2.1 Literature search

To compile clinical trial results for first-aid methods in treating *Carybdea* spp. envenoming and species distributions, we searched digitised and undigitised material. Keywords used for the digitised literature search included, along with known variant spellings of the genus and species, the following: *Carybdea*, *Charybdea*, *rastonii*, *rastoni*, jimble, envenomation, sting, and first aid. English keywords were selected for practicality. All results were considered due to the limited information available.

Databases searched included Google Scholar, PubMed, Trove and university library databases. Undigitised archival documents from the Dr R. V. Southcott collection held in the South Australian State Library were reviewed to determine the relevant histories of carybdeid taxonomy and envenomations and his extensive research into cubomedusa species. Source documents were reviewed for mentions of *C. rastonii* taxonomy, distribution, and references to museum specimens and clinical symptoms. Where authors’ species spelling in literature differs from the currently accepted binomial we list the original spelling in supplementary materials Table S1.

### 2.2 Museum material examined

We examined museum specimens identified as ***Alatina* sp.** (syn. *Carybdea alata*)***, Carybdea paragrandis*** (nomen nudum), ***C*. *rastonii*** (or variant spellings)***, C. xaymacana*,** and ***Carybdea* sp.** housed in the collections of the South Australian Museum (SAM), Queensland Museum Kurilpa (QMK) and Queensland Museum Tropics (QMT), Australia. Preserved specimens were examined under a stereo light microscope. Permanent histological slides of *C. rastonii* were examined under ×1000 magnification with a compound microscope. Full details of materials examined can be found in the supplementary materials (Data S1).

## 3 Results

### 3.1 Carybdea clinical symptoms

Clinical reports of *Carybdea rastonii* envenomation symptoms range in severity from a moderately painful stinging sensation (Rowland and Eipper, 2018; Southcott, 1982), pain lasting for 2 h (Cegolon et al., 2013), or injuries resembling burns with pain continuing over 24 h (pers. comms. L. Simeoni, 2025). *Carybdea rastonii* envenoming is not known to cause severe long-term effects (Southcott, 1982; Tibballs, 2006; Tibballs et al., 2012).

*Carybdea rastonii* stings are often characterised by up to four linear lesions around 100 mm long, corresponding to the number and length of the tentacles that come in contact with the casualty, or weals a few millimetres across that may last a few days (Tibballs, 2006). Histology conducted on skin lesions, post a suspected *C. rastonii* tentacle envenomation, on a casualty's forearm showed symptoms corresponding with allergic contact dermatitis and pain that persisted between 10 min to 8 h (Ohtaki et al., 1990). It is now thought that the species in Ohtaki et al. (1990) would most likely be *C. brevipedalia* Kishinouye, 1891, and not *C. rastonii*, based on species distribution changes (Acevedo et al., 2019; Bentlage et al., 2010).

### 3.2 Jellyfish first aid

Reports of jellyfish blooms and associated hazards have increased globally in recent decades (Duarte et al., 2013; Lee et al., 2023). Surf Life Saving Australia (2024) reported 23,371 cases of first-aid treatment performed across Australia due to marine stingers within a 12-month period (2023–2024). In South Australia, almost ¼ (153 of 550) of all beachside first aid was in response to a marine envenoming. In 2024, an estimated 1.2 million people visited South Australian coasts (Surf Life Saving Australia, 2024), highlighting the importance of increasing our knowledge of local jellyfish ecology, behaviour, and life cycles to help inform first aid treatment and support public health announcements.

For the treatment of these potential hazards, the Australian Resuscitation Council (ARC) is responsible for setting first-aid treatment guidelines in Australia. There are inconsistencies between ARC recommendations and those of marine stinger first-aid guidelines produced by peak bodies (e.g. Surf Life Saving Australia) (Supplementary material Table S2). Due to variations in how different species may react to treatments, it is not feasible to provide a single, general first-aid recommendation for all jellyfish envenomations (ANZCOR, 2025).

The ARC divides its two major jellyfish first-aid procedures into tropical and non-tropical regions. For non-tropical regions, such as southern Australia, they recommend removing any tentacles and then immersing the sting site in hot water for 20 min. The ARC does not directly address any protocol for treating *Carybdea rastonii* specifically (ANZCOR, 2025).

Surf Life Saving Australia's jellyfish first-aid guidelines for jimble stings recommend removing any tentacles, washing the area with seawater, submerging the afflicted area in hot water for 20 min, and applying a cold pack if previous treatments are ineffective (Surf Life Saving Australia, 2020). Surf Life Saving – South Australia recommends removing tentacles with seawater or picking off with fingers, rinsing with vinegar, and applying a cold pack (Surf Life Saving South Australia, 2025). St John Ambulance Australia (2022) advises a vinegar rinse followed by the application of a cold pack in the case of jimble sting.

Journal articles and textbook-specific first-aid treatment recommendations for *Carybdea rastonii* envenomation include applying a cold pack or ice for 5–10 min, for local pain management (Exton et al., 1989; Williamson et al., 1996) or to perform a vinegar rinse on the envenomation site (Newman-Martin, 2007). Application of a local anaesthetic may be warranted when lesions are extensive (Clinical Toxinology Resources, 2025; Newman-Martin, 2007; Tibballs, 2006). Despite first-aid treatment recommendations, no publications were found pertaining to *C*. *rastonii* envenomation pain treatment experiments.

Table 1 summarises experiments for pain management post an envenomation event from jellyfish belonging to the *Carybdea* genus (those considered low health risk). The jellyfish *Carybdea alata* is synonymised with the *Alatina* genus and is referred to as such in Table 1 and in addition it is thought that there may be more than one species and so we refer to those as *Alatina* sp. (Gershwin, 2005).

**Table 1:** Published clinical experiments for treating pain from carybdeid envenomation, detailed along with valid scientific names. Original published scientific names are detailed in Table S1.

Treatment	Species	Location	Results	Notes	Cohort size	Author
**Hot water immersion**	*Carybdea* spp.	Geographe Bay, Western Australia	88% pain relief	4 stings per participant. Temp. 45 °C. Unspecified pain scale	5	Taylor (2007)
	*Alatina* sp.	Honolulu Harbor, USA	Effective compared to vinegar or papain	2 stings, 2 treatments per participant. Temp. 40-41 °C	25	Nomura et al. (2002)
**Hot pack**	*Alatina* sp.	Waikiki Beach, USA	Some effect on pain compared to control, not clinically significant	Vinegar rinse before pain treatment. In-field first aid. Temp. 43 °C	127	Thomas et al. (2001b)
**Cold pack**	*Alatina* sp.	Waikiki Beach, USA	Some effect on pain compared to control, not clinically significant	Vinegar rinse before pain treatment. In-field first aid. Temp. 5 °C	127	Thomas et al. (2001b)
**Ice**	*Carybdea* spp.	Geographe Bay, Western Australia	Unsuccessful in relieving pain	4 stings per participant. Unspecified pain scale	5	Taylor (2007)
**Vinegar**	*Carybdea* spp.	Geographe Bay, Western Australia	Unsuccessful in relieving pain	4 stings per participant. Unspecified pain scale	5	Taylor (2007)
**Aluminium sulphate**	*Carybdea* spp.	Geographe Bay, Western Australia	Unsuccessful in relieving pain	4 stings on each of the 5 participants to test 4 treatments	5	Taylor (2007)
**Aluminium sulphate (Sting-Aid)**	*Alatina* sp.	Waikiki Beach, USA	Not clinically significant compared to control	Seawater control	62	Thomas et al. (2001a)
**Papain (<1% as a meat tenderiser)**	*Alatina* sp.	Waikiki Beach, USA	Not clinically significant compared to control	Seawater control	62	Thomas et al. (2001a)
**Freshwater**	*Alatina* sp.	Waikiki Beach, USA	Not clinically significant compared to control	Seawater control	62	Thomas et al. (2001a)

In our search, we included *in vitro* investigations for the prevention of *Carybdea rastonii* nematocysts firing, to prevent further envenoming. One significant study by Fenner and Williamson (1987) investigated the efficacy of different theoretical treatments on specimens they identified as *C. rastonii*. The authors found that vinegar, baking soda, and Stingose® (active ingredient aluminium sulphate) were all effective, to a similar extent, for inhibiting unfired nematocysts in a Petri dish. Additionally, they found that methylated spirits were likely to encourage nematocyst firing, a previously favoured treatment in the tropics. Ballesteros et al. (2021) also found that vinegar is effective in inhibiting the firing of *C. marsupialis* nematocysts.

The ARC states that there is “considerable confusion” about the correct first-aid measures for jellyfish stings (Bridgewater, 2007), and it has been remarked upon in the literature that there is a conspicuous gap in jellyfish first-aid knowledge (Little, 2008; Seymour, 2017). Anecdotal information regarding the treatment of *Carybdea rastonii* stings suggests that common first-aid treatments for jellyfish stings (hot water, cold packs, Stingose® or vinegar) are ineffective when used on *C*. *rastonii* stings (pers. comms. J. Baker, 2024; pers. comms. L. Simeoni, 2025).

### 3.3 Binomial nomenclature and etymology

The genus name, *Carybdea,* was established by Péron and Lesueur (1809) and used through to Acevedo et al. (2019). The variant spelling, *Charybdea*, was listed by Agassiz (1846) and used sporadically by authors (Claus, 1878; Haacke, 1886; Haeckel, 1879; Mayer, 1910) until *Carybdea* was re-established by Kramp (1961). *Carybdea rastoni* was a common spelling (Kramp, 1961; Southcott, 1967) until the original spelling of *rastonii* regained widespread use (Acevedo et al., 2019; Gershwin, 2006; Matsumoto, 1995). The International Code of Zoological Nomenclature (ICZN) (1999) Mandatory Article 33.4 states that species-group names where the original spelling had a suffix -ii should be preserved over a subsequent spelling with the suffix -i. Article 32.5 also states that errors in Latinization should not be considered as incorrect spelling. Accordingly, we conclude that the correct nomenclature is *Carybdea rastonii*.

Common names found for *Carybdea rastonii* include: small box jellyfish (Newman-Martin, 2007), jimble (Australia), sea wasp (USA), mona, andonkurage, lantern medusa (Japan) (Halstead, 1965) and Raston's box jelly (Coleman, 1999). Today's common name of “jimble” for *Carybdea rastonii* in Australia can be traced back to South Australian Zoologist, Dr R. V. Southcott, who wrote in his unpublished letter, *“*Carybdea rastoni ‘*jimble’. This is the word used for this species in the Southcott household, being purely a made-up word, and used for some years in an unpublished children's story I tell my kids … The name is no doubt ultimately traceable to Edward Lear and Lewis Carroll … ‘gimble’ … ‘gyre’ … ‘jumbly’”* (SLSA: PRG 233/9.10 (L213-214), 1963).

### 3.4 Cnidae identification informing clinical treatment

The presence or absence of cnidae classes (the cnidom) is useful at higher taxonomic levels (e.g. order). The cnidae class of nematocysts, in particular, may assist with identification at lower taxonomic levels (e.g. genus, species) (Fautin, 2009). Cubomedusae have three main categories of nematocyst: euryteles, isorhizas and mastigophores (Gershwin, 2006).

The use of nematocyst morphology in the taxonomic process has long been accepted by sea anemone taxonomists (England, 1991; Forero-Mejia et al., 2024; Weill, 1934), but less commonly included in jellyfish species descriptions of Carybdeida (Yasanga et al., 2024). Previously, Gershwin (2006) suggested that the cubozoan cnidom may not be relevant to taxonomy, though it is important to document. Recently, more researchers have seen the value in publishing the cnidom of jellyfish, though it is still not standard practice (Gershwin, 2006; Yasanga et al., 2024, 2025). The most recent taxonomic review of *Carybdea rastonii* (Acevedo et al., 2019) did not include a description of nematocysts from specimens examined.

Skin scraping, a technique of removing nematocysts from the sting site of an envenomed casualty, has been used by clinicians to facilitate causative jellyfish identification, to aid in informing treatment (Halstead, 1965; Huynh et al., 2003). Researchers have had some success with identifying jellyfish via skin scrapings, such as in the case of *Carukia barnesi* (Huynh et al., 2003) and *Chironex fleckeri* envenoming (Currie and Wood, 1995).

Current methods and success of acquiring skin scraping samples vary; there is no standard technique or training in place for clinicians to perform skin scrapings, and no available nematocyst skin scraping reference guide for identifying species. These techniques have potential for use in guiding clinical diagnoses yet should be used with caution until there is more knowledge of individual jellyfish cnidoms (Halstead, 1965).

### 3.5 Carybdea geographical distribution

Haacke (1886) described the type specimen of *Carybdea rastonii* from St. Vincent's Gulf, South Australia. Subsequent observations of *C. rastonii* within South Australia were made by Mr F. J. Mitchell at Flagstaff Landing, near Streaky Bay, Eyre Peninsula, Stokes Bay, Kangaroo Island and St. Vincent's Gulf (Cleland and Southcott, 1965; SLSA: PRG 233/9.10, 1958). *Carybdea rastonii* was thought to occur throughout the tropical Pacific, Malaysia, Marquesa, Hawai'i and southern California, United States of America (Halstead, 1965), southern Philippines, Indonesia (Clinical Toxinology Resources, 2025). Within Australia, the reported distribution is across the southern coastal waters of Australia (Clinical Toxinology Resources, 2025; Coleman, 1999; Rowland and Eipper, 2018; Southcott, 1982), Western Australia (Coleman, 1999; Rowland and Eipper, 2018), New South Wales (Coleman, 1999), and Queensland (Rowland and Eipper, 2018).

**Fig. 1:**
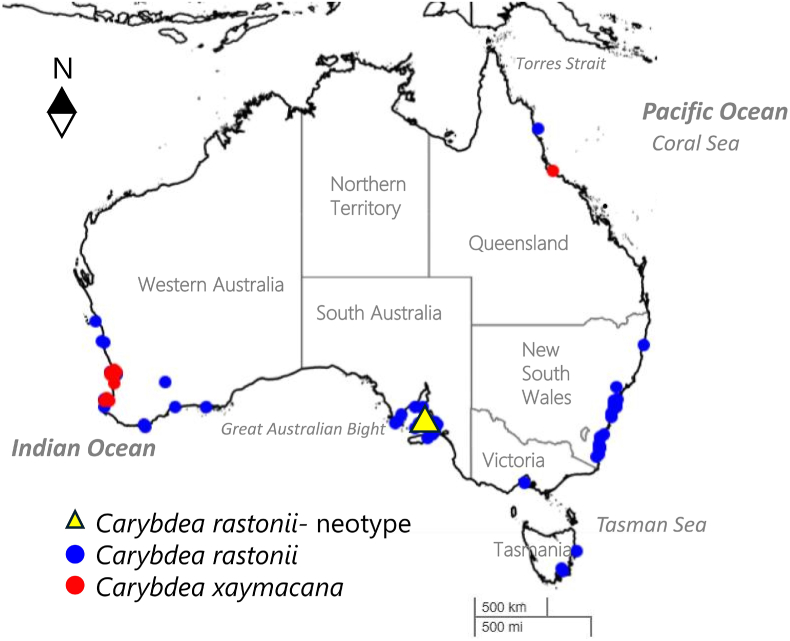
Current distribution of *Carybdea* spp. based on museum and human observation records. Occurrence records (175) of *Carybdea rastonii* (blue), *Carybdea rastonii* neotype (yellow) and *Carybdea xaymacana* (red) in Australia. Georeferenced data generated from Atlas of Living Australia, Ala.org.au (accessed 16 May 2025).

There have been several revisions of the accepted distribution of *Carybdea rastonii*. What was initially thought to be *C. rastonii* in Southern California is confirmed as *C. confusa* Straehler-Pohl, Matsumoto & Acevedo, 2017. Jellyfish previously identified as *C. rastonii* in Hawai'i and Japan were revised to *C. arborifera* Maas, 1897 (Hawai'i) and *C. brevipedalia* (Japan), respectively (Acevedo et al., 2019; Bentlage et al., 2010). The current accepted distribution of *C. rastonii* has been reduced to the coastline of Australia (Fig. 1) (Acevedo et al., 2019; Karunarathne and de Croos, 2020).

### 3.6 Museum material

**Fig. 2:**
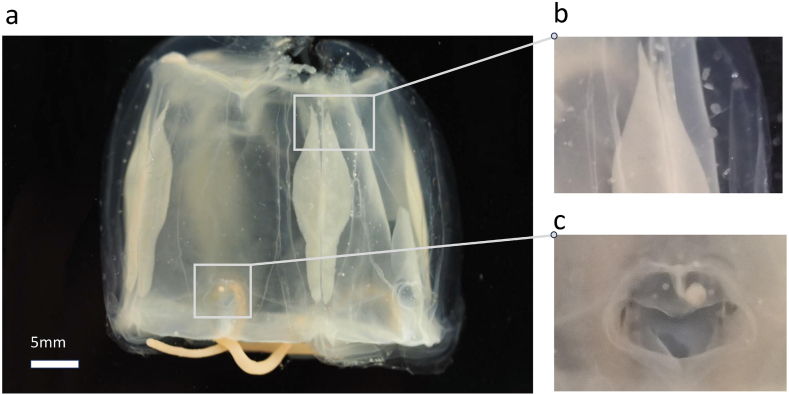
*Carybdea rastonii* preserved specimen photographed at the South Australia Museum (Reg No: SAM H1624). **a**. full body view. **b.** characteristic white “stinging warts” on the translucent exumbrellar surface, **c.** heart-shaped rhopaliar niche ostium. Additional photography in supplementary materials (Fig. S1 and S2). Photo: N. Meyler.

Specimens examined were collected from South Australia, Western Australia, Queensland and the Comoros Isles, Mozambique Channel. The type locality of *Carybdea rastonii* is the Gulf of St. Vincent, South Australia. Images of the designated neotype for *C. rastonii* can be seen in Fig. 2. Key morphology characteristics are 4 single tentacles (one per pedalium) (Fig. 2a), small white “stinging warts” on the translucent exumbrellar surface (Fig. 2b) and heart-shaped rhopaliar niche ostia (Fig. 2c). *Carybdea rastonii* can be diagnosed by a rounded knee-bend on each pedalium, and triforked velarial canals (Haacke, 1886).

One registered specimen (QMT G55282) is labelled as a holotype and publicly available online as *Carybdea paragrandis* (The State of Queensland (Queensland Museum), 2026). As no published description exists for this specimen and the name having entered the public domain it becomes nomen nudem (naked name). The specimen in question clearly differs morphologically from *C. rastonii* and *C. xaymacana* and accordingly requires a full taxonomic description.

## 4 Discussion

Based on the knowledge available, we identify the two primary goals of *Carybdea rastonii* first-aid treatment are to;

1. Prevent further discharge of venom-containing nematocysts into the casualty.
2. Minimise ongoing pain.

Our search of available literature did not reveal any effective, evidence-based first-aid treatments for *Carybdea rastonii* envenoming, a jellyfish occurring in southern Australia, which can produce painful weals in casualties. Across all studies, the application of heat or hot water immersion is reported as the treatment with the most potential for clinical importance in *Carybdea* envenomation events*,* though these findings are minimal and inconclusive (Nomura et al., 2002; Thomas et al., 2001a, 2001b).

The ARC recommends applying a cold pack for jellyfish stings (ANZCOR, 2025). Though no evidence-based research was found to support cold packs nor hot water as a clinically effective treatment for *Carybdea rastonii* stings and pains. The lack of any rigorous experimentation of *C. rastonii* envenomation treatment protocols is compounded by the presence of anecdotal information that popular pain management recommendations are ineffective on jimble stings (pers. comms. J. Baker, 2024; pers. comms. L. Simeoni, 2025), revealing a need for robust, evidence-based research to inform future first-aid treatment for *C. rastonii*.

Few of the suggested treatments for jellyfish stings can be traced back to evidence-based clinical trials that support their use, and no studies relevant to the *Carybdea* genus were robust enough to demonstrate clinical significance. Pain relief treatment studies were not performed using *Carybdea rastonii,* and many of the jellyfish species trialled in the gathered clinical data for *Carybdea* are no longer considered part of the *Carybdea* genus after taxonomic revision (see Table 1). Several studies were performed on *Alatina* sp. or what the researchers determined were *Carybdea alata* (a now synonymised species placed in the genus *Alatina*) (Nomura et al., 2002; Thomas et al., 2001a, 2001b). This highlights the value of clarifying taxonomic descriptions and distribution for small box jellyfish in the context of evaluating first-aid methods for evidence-based studies.

Fenner and Williamson (1987) did not include specimens of *Carybdea rastonii* from its type locality in South Australia nor lodge any specimens with museums for future species verification, making their experiment unverifiable. Methods to prevent firing of nematocysts after stinging are often species-specific (Williamson et al., 1996), increasing the importance of proper taxonomic identification of *C. rastonii*. One source recommended removing cubozoan tentacles from a sting site using bare fingertips if no gloves were available (Williamson et al., 1996), a recommendation that has been perpetuated through the Australian Resuscitation Council that sets the standard for Australian for first-aid providers (ANZCOR, 2025; Surf Life Saving South Australia, 2025). However, removing any cnidarian tentacles without protection may cause further undue harm. This is exemplified in a case where a tentacle was removed by the sting casualty, resulting in extensive, ongoing issues (de Freitas et al., 1995).

No research has been conducted to investigate the most effective method of removing *Carybdea rastonii* tentacles and there is limited research available on this for jellyfish in general. The common recommendation is to use gloves or tweezers when removing tentacles from a sting casualty to reduce risk of secondary stings (Cegolon et al., 2013; Remigante et al., 2018; Surf Life Saving Australia, 2024), glove thickness (e.g. nitrile vs latex) may be a consideration due to the ability of nematocysts to penetrate hard-bodied prey (Park et al., 2017).

The practicality of proposed treatments must be considered in the context of the envenomation encounter. One group susceptible to jellyfish stings are SCUBA divers (Suriyan et al., 2019), who, when using exposure suits, are most exposed on their head (excluding eyes where a mask would be worn) and neck (Burnett et al., 1994). This makes divers poor candidates for submerging the sting site for long periods (>20 min), i.e. submerging neck or face, and would likely require access to a ready supply of hot water (45 °C). Hot water immersion and hot showers pose a potential threat to the health of SCUBA divers, as it is commonly recommended to avoid rapid thermal stresses after surfacing due to the increased risk of decompression sickness (Lippmann, 2024; Pollock, 2020). In addition, first aid is most effective on-site at the time of the incident, and providing hot water to beachside first responders is strategically challenging, particularly while at sea or in remote/rural regions.

Identifying causative jellyfish is crucial to the practical application of evidence-based first-aid methods following envenomation, and accurate naming is essential for disseminating appropriate information for public health. It has yet to be determined if common names used in association with *Carybdea rastonii* link directly to a specific or multiple species.

From our review of *Carybdea rastonii* material in southern Australian collections, it is evident that there are multiple species of *Carybdea* in the region, and some are likely to be taxonomically documented as new species. Much of the confusion behind the distribution range of *C. rastonii* stems from a lack of rigorous identification and the previous belief that all *Carybdea* in Australia were *C. rastonii* only (Cleland and Southcott, 1965). The lack of clarity regarding species, their distribution, and correlation to symptoms based on only a few envenomation records has created a knowledge gap.

Based on our examination of museum specimens, we concur with Gershwin (2005) and Acevedo et al. (2019) that there is more than one species of *Carybdea* in Australian waters. The stinging warts (Fig. 2b) first described by Haacke (1886) are a good candidate for assessment as a key morphology characteristic in diagnosing the species and clarifying taxonomic boundaries along with a comparative study of cnidae in the species. As cnidae, particularly nematocysts are the organelles that facilitate toxins entering the body, they may be the link between our knowledge of jellyfish and the symptoms its venom can cause (Peng et al., 2024). The nature of the connection between the cnidom, the contained venom, and the subsequent envenomation symptoms is not yet fully understood for *Carybdea* species to facilitate appropriate evidence-based first aid protocols.

Revisions of *Carybdea* taxonomy (Larson and Arneson, 1990; Stiasny, 1922; Straehler-Pohl et al., 2017) highlight that existing envenomation records and treatment studies for some jellyfish are likely to be based on species misidentification. Compounded with misidentification of species are incidents where locality coordinates are mismatched with locality names, creating distribution uncertainty (Acevedo et al., 2019; Straehler-Pohl et al., 2017). Gershwin (2005) suggests that identification of specimens from outside of Australia as *C. rastonii* should be viewed cautiously. Misidentifications and locality confusion that do not accurately reflect the true geographic ranges of jellyfish, as demonstrated with *C. rastonii*, have implications for jellyfish treatment guidelines, which are currently based on geographic location.

There is a tangled web of misidentified *Carybdea* specimens across Australia and potentially globally. With so few specimens of southern Australian cubozoans properly preserved and registered in museum collections, carybdeid taxonomy and first-aid protocols cannot progress without additional research. There is a clear need for a detailed revision of the species and description of new species throughout Australia, as well as a need to link species to clinical symptoms and range distributions for conclusive evidence-based first-aid studies.

Future *C*. *rastonii* research will impact first-aid treatment, public health, tourism, ecology and management of cnidarian envenoming. In addition, we did not locate any documented Aboriginal and Torres Strait Islander knowledge pertaining to jellyfish in South Australia and this may prove to be valuable research avenue. We reiterate the call for evidence-based first-aid research and treatments for jellyfish stings.

## 5 Conclusion

Current literature shows that the emerging trend of a one-size-fits-all first-aid protocol based on geographic location is not appropriate for treating cnidaria envenoming. Establishing the correct scientific name, distribution, and identification for venomous animals is crucial for efficiently communicating and reviewing information regarding clinical symptoms and treatment documentation for public health. Our review of the jimble, *C*. *rastonii*, solidifies taxonomic nomenclature, highlights misspellings, and establishes the correct spelling. We summarise the compounding confusion regarding first-aid response to Cubozoan envenomation, especially for treatment based on geographic locality.

We have found an abundance of unverifiable carybdeid first-aid treatments and have emphasised the lack of any evidence-based clinical trials investigating treatments for *C*. *rastonii* envenomation. First aid on-site is the most crucial step in minimising symptoms. The question remains: what first-aid method is effective for treating *C. rastonii* envenoming?

## CRediT authorship contribution statement

**N.E. Meyler:** Writing – review & editing, Writing – original draft, Investigation, Data curation. **M.L. Mitchell:** Writing – review & editing, Supervision, Methodology, Investigation, Conceptualization.

## Funding

This research received no specific grant from funding agencies in the public, commercial, or not-for-profit sectors.

## Declaration of competing interest

The authors declare that they have no known competing financial interests or personal relationships that could have appeared to influence the work reported in this paper.

## Data Availability

No data was used for the research described in the article.
