# Supplementary material for: The ‘jimble’, a southern Australia box jellyfish, Carybdea rastonii Haacke, 1886: clinical symptoms, first-aid treatments and species distribution
Source: Toxicon X. 2026 Apr 1;30:100254. doi: 10.1016/j.toxcx.2026.100254 (PMC13126026; doi:10.1016/j.toxcx.2026.100254)
Supplement: Multimedia component 1 [file mmc1.docx]

**The ‘jimble’, a southern Australia box jellyfish, *Carybdea rastonii* Haacke, 1886: clinical symptoms, first-aid treatments and species distribution**

Meyler, N. E.^1#^, Mitchell, M. L. ^1, 2#^

^1^Toxinology Department, Women's and Children's Health Network, North Adelaide, South Australia 5006, Australia

^2^School of Medicine, College of Health, Adelaide University, Adelaide, South Australia, 5005, Australia

#Correspondence to: Michela L. Mitchell [michela.mitchell@sa.gov.au,](mailto:michela.mitchell@sa.gov.au,) Niamh E. Meyler nime5024@gmail.com

ORCID:

Niamh E. Meyler: <https://orcid.org/0009-0003-2897-8158>

Michela L. Mitchell: <https://orcid.org/0000-0001-6331-534X>

**Keywords:** Marine envenomation, Cnidaria, Toxinology, Public health, Epidemiology, Dive hazards

**Table S1:** Comprehensive synonymy of *Carybdea* within the literature featured in this publication.

| **Accepted nomenclature** | **Synonyms** | **Reference** |
| --- | --- | --- |
| *Carybdea rastonii* | *Carybdea rastoni* | Cleland and Southcott, 1965 |
|  |  | Halstead, 1965 |
|  |  | Southcott, 1963, 1967 |
|  |  | Fenner and Williamson, 1987 |
|  |  | Williamson et al., 1996 |
|  |  | Coleman, 1999 |
|  |  | Tibballs, 2006 |
|  |  | Newman-Martin, 2007 |
|  |  | Cegolon et al., 2013 |
|  |  | Clinical Toxinology Resources, 2025 |
|  |  |  |
|  | *Charybdea rastonii* | Haacke, 1886 |
|  |  |  |
| *Carybdea brevipedalia* | *Carybdea rastoni* | Ohtaki et al., 1990 |
|  |  |  |
| *Alatina* sp. | *Carybdea alata* | Nomura et al., 2002 |
|  |  | Thomas et al., 2001a, 2001b |

**Table S2:** Current recommendations for first-aid treatment from Australian aid providers in response to non-tropical jellyfish and/or jimble envenomation. +=recommended step in first aid

| **First-aid organisations** | **Jellyfish group responsible for envenomation** | **Vinegar** | **Hot water immersion** | **Cold pack** | **Seawater** |
| --- | --- | --- | --- | --- | --- |
| **Australian Red Cross** | Non-tropical jellyfish |  | **+** | **+** | **+** |
|  |  |  |  |  |  |
| **ANZCOR Australian and New Zealand Committee on Resuscitation** | Non-tropical jellyfish |  | **+** | **+** | **+** |
|  |  |  |  |  |  |
| **Health Direct** | Jimble (other jellyfish not incl. bluebottle or major box jellyfish) |  | **+** |  | **+** |
|  |  |  |  |  |  |
| **St John Ambulance** | Non-tropical jellyfish |  | **+** | **+** |  |
|  | Jimble | **+** |  | **+** |  |
|  |  |  |  |  |  |
| **Surf Life Saving Australia** | Jimble |  | **+** | **+** | **+** |
|  |  |  |  |  |  |
| **Surf Life Saving Queensland** | Jimble |  | **+** | **+** | **+** |
|  |  |  |  |  |  |
| **Surf Life Saving South Australia** | Jimble | **+** |  | **+** | **+** |
|  |  |  |  |  |  |
| **Queensland Poisons Information Centre** | "Other" jellyfish (not incl. bluebottle, tropical box jellyfish) |  | **+** |  | **+** |

**Data S1** Systematics and material examined data

**Abbreviations**

Coll: collector

Det: determined

SAM – South Australia Museum,

OMK – Queensland Museum Kurilpa

QMT – Queensland Museum Tropics

RVS – Dr. Ronald V. Southcott

Unreg. – Unregistered

**Class Cubozoa Werner, 1973**

**Order Carybdeida Gegenbaur, 1857**

**Family Alatinidae Gershwin, 2005**

**Genus *Alatina* Gershwin, 2005**

***Alatina* sp.**

Synonymy cf. *Carybdea alata* Reynaud, 1830

**Material examined:** *Africa, Mozambique channel.* **QMK G317053,** Off Moroni, Comoros Isles, 11°40'0.0012''S, 43°16'0.0012' E, Det: L. Gershwin, 01 Jan 05, 70% ethanol, 2 specimens. Label notes: Peter Fenner collection, identified *Carybdea alata*. *Indonesia,* *Kau Bay*. **SAM** unreg., Halmahera Island, 1°20'N, 128°1'E, Coll. TE VEGA expedition students, 24 Sep 1963, Det. J. Bennets, <10 % formalin, 1< specimens.

Comments: QMK G317053, Specimens in a deteriorated condition having been stored in ethanol.

**Family Carybdeidae Gegenbaur, 1857**

**Genus Carybdea Péron & Leseur, 1809**

***Carybdea rastonii* Haacke, 1886**

See table S1 for synonymy

**Type Locality:** Gulf of St Vincent, Australia

**Type material examined:**  *Australia, South* *Australia.* **SAM H1624,** [Neotype] Waterloo Bay, 22 Feb 1999, L. Gershwin, <10 % formalin, 1 specimen.

**Additional material examined:** *Australia, South Australia.* **SAM,** unreg., Kingston Park (~100 yds from shore), Gulf of St Vincent, 04 Feb 1962, Coll. S. A. Shepherd, Det: R.V. Southcott, <10 % formalin, 1< specimens, Label notes: RVS-A555D *Locality Unknown.* **SAM** **H3509,** [Histology slide] 21 Mar 1963, Coll. S. A. Shepherd, RVS. Label notes: (RVS Id: A661B,C) section of gonad.

Comments: SAM H1624, heart-shaped rhopaliar niche ostia, triforked velarial canals, white warts on exumbrellar surface. SAM unreg., RVS A555D experimental, heart-shaped rhopaliar niche ostia, not as deep-set as *C. rastonii* neotype, distinctive bands on tentacles due to retraction. SAM H3509, slides have dried out and require conservation to be able to view the original histology, including any cnidae.

***Carybdea paragrandis* (nomen nudum)**

**Material Examined:** *Queensland, Australia.* **QMT G55282**, Moore Reef, Northeast Queensland, 16º52'0.01" S 146º13'59.99" E, Coll. L. Gershwin, Det. L. Gershwin, <10 % formalin.

Comments: QMT G55282, listed in the Queensland Museum online collections as a holotype.

***Carybdea xaymacana* Conant, 1897**

**Type Locality:** Fort Henderson, Kingston Harbor, Jamaica.

**Material Examined** *Australia,* *Queensland.* **SAM H918**, Cairns, Palm Cove, 27 Dec 1997, Coll. J+G Seymour, June 1999, Det. L Gershwin, <10 % formalin, 1 specimen. *Western Australia.*  **QMK G317044,** Cottesloe Beach, Perth, 31º58'60"S 115º45'0"E, 18 Apr 1987, Det. L. Gershwin, <10 % formalin. Label notes: identified *C. rastoni*

Comments: SAM H918, Heart-shaped rhopaliar niche ostia, single tentacle per pedalium (4), white nematocyst warts, lemon drop eurytele type and p-mastigophore. QMK G317044, cannot conclude to be *C. xaymacana*., previously identified as *C. rastoni* Peter Fenner collection.

***Carybdea* sp.**

**Material Examined:** *Australia, Queensland.* **QMK G329973,** Sandgate Beach, Brisbane 27°18'00.0"S 153°04'00.0"E, 7 Feb 2009, L. Jeays, Det. Dr M. Ekins, <10 % formalin; *New South Wales.* **SAM** unreg., Lake Illawarra, 1-6 June 1964, Coll. Isobel Bennett, <10 % formalin. Label note: RVS 800; *Western Australia*. **SAM H1292,** Fremantle, 32°3'S 115°44'E, 9 Dec 2000, Coll. W. Zeidler & L. Gershwin, <10 % formalin. Label notes: likely juvenile *C. xaymacana*.

Comments: QMK G329973 multiple tentacles 3+ per pedalium.

**Specimen Differential:** Comparison of specimens to the purportedly designated neotype for *Carybdea rastonii* reveals that some specimens lack characteristics diagnostic of the species; for example, those without exumbrellar warts or with a shallow-inset heart-shaped can clearly be discerned from non-*C. rastonii* specimens. Specimens of *C. rastonii* have discernible, heart-shaped rhopaliar niche ostia, triforked velarial canal, and the presence of nematocyst warts. Specimen QMK G329973 had multiple tentacles on each pedalium but was identified as *Carybdea* sp. a clear misidentification. Specimens SAM H918 and QMK G317044 are consistent with the *Carybdea* genus but require further comparison to the *C. xaymacana* type specimen. Analysis has revealed that several C*arybdea* specimens in Australian museums are currently misidentified and require further taxonomic work.


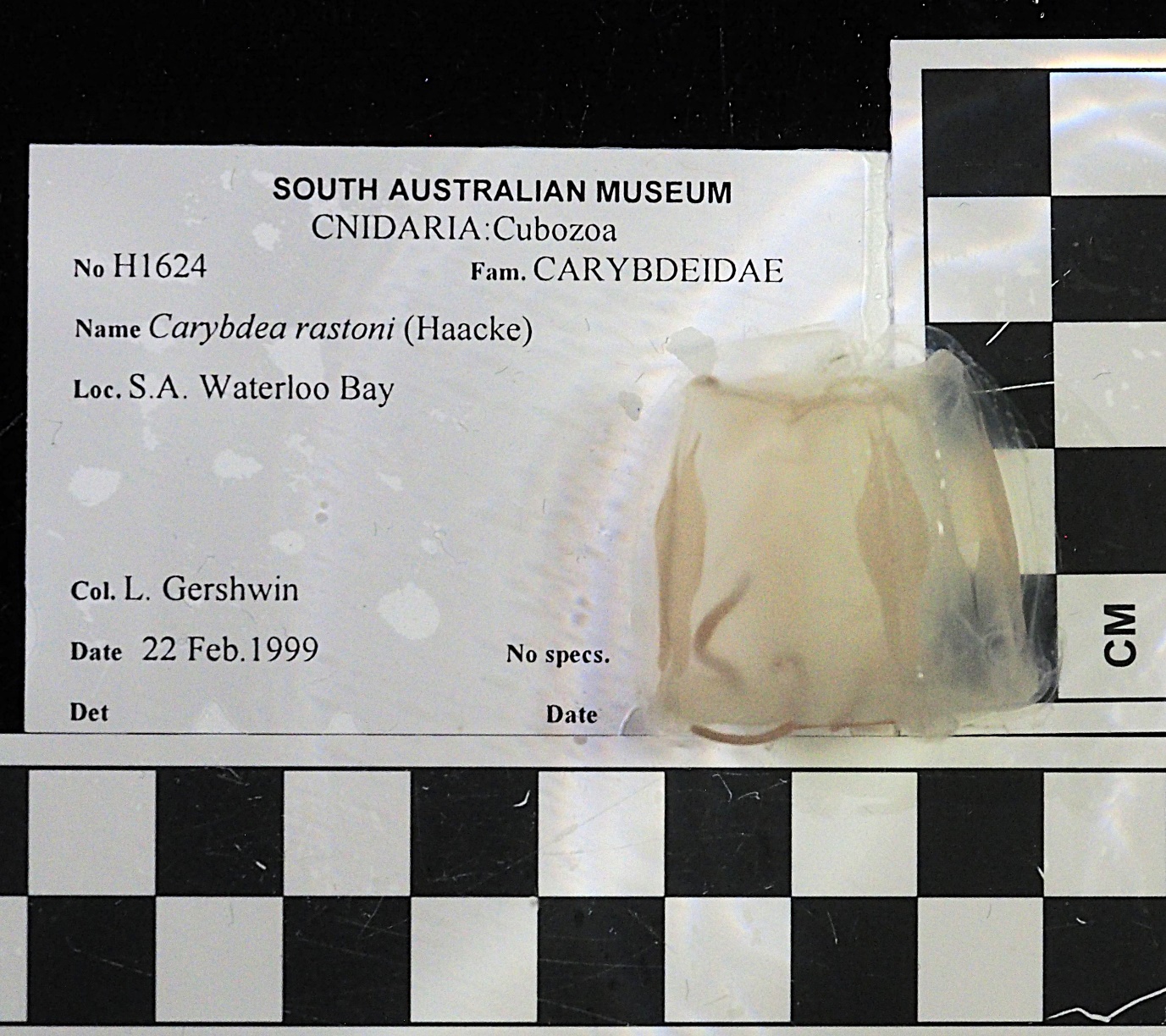


**Fig. S1.** *Carybdea rastonii* (syn *C. rastoni*) SAM H1624, preserved neotype collected from Waterloo Bay, South Australia. Photographed showing specimen label and scale bar at the South Australia Museum (SAM). Photo: N. Meyler.


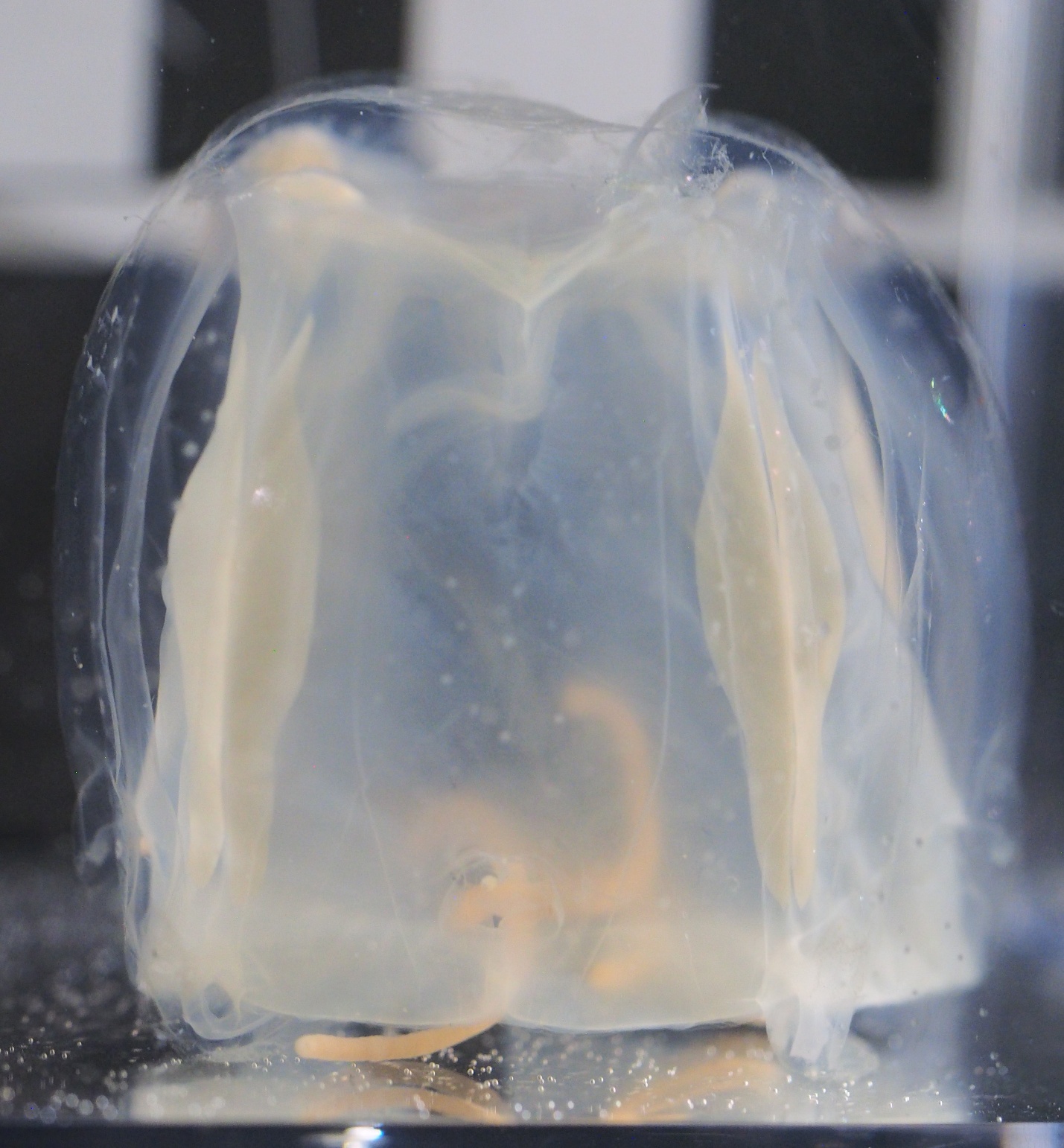


**Fig. S2.** *Carybdea rastonii* (syn *C. rastoni*) SAM H1624, preserved neotype collected from Waterloo Bay, South Australia. Photographed showing the small white warts (arrow) indicative of the species at the South Australia Museum (SAM). Photo: N. Meyler.

**References**

ANZCOR Australian and New Zealand Committee on Resuscitation, 2025. Guideline 9.4.5 - Envenomation - Jellyfish Stings [WWW Document], (accessed 1.4.24)

Australian Red Cross, 2025. First Aid for a jellyfish sting, [WWW Document]. URL <https://www.redcross.org.au/firstaid/basics/jellyfish/> (accessed 7.8.25)

Cegolon, L., Heymann, W.C., Lange, J.H., Mastrangelo, G., 2013. Jellyfish stings and their management: A review. Mar. Drugs. 11, 523-550. 10.3390/md11020523

Cleland, J.B., Southcott, R. V., 1965. Injuries to man from marine invertebrates in the Australian region. Commonwealth of Australia, Canberra.

Clinical Toxinology Resources, [WWW Document], 2025. URL <http://toxinology.com/fusebox.cfm?fuseaction=main.marine_invertebrates.results&Common_Names_term=jimble&Phylum_term=&Class_term=&SubClass_term=&ord_term=&Genus_term=&Species_term=&countries_terms=&region_terms=&General_Information__term=> (accessed 3.7.25)

Coleman, N., 1999. Dangerous sea creatures. Neville Coleman’s Underwater Geographic Pty. Ltd.

Conant, F.S., 1897. The cubomedusae. Johns Hopkins University Circulars 4, 1, 3–22.

Fenner, P.J., Williamson, J., 1987. Experiments with the nematocysts of *Carybdea rastoni* (“Jimble”). Med. J. Aust. 147, 259–259. 10.5694/j.1326-5377.1987.tb133433.x

Gegenbaur, C., 1857. Versuch eines systemes der medusen, mit beschreibung‚ neuer der wenig gekannter formen; zugleich ein beitrag zur kenniniss der fauna des Mitielmeeres. Zeitschrift für wissenschaftliche Zoologie 209–230.

Gershwin, L., 2005. Taxonomy and phylogeny of Australian cubozoa [doctoral dissertation]. James Cook University, URL <http://eprints.jcu.edu.au/27395/> (accessed 14.2.25)

Haacke, W., 1886. Die scyphomedusan des St. Vincent Golfes. Jenaische Zeitschrift Für Naturwissenschaft 20, 588–638.

Halstead, B.W., 1965. Poisonous and venomous marine animals of the world, The Darwin Press, Inc. .

Health Direct, 2024. Jellyfish stings, [WWW Document]. URL [https://www.healthdirect.gov.au/jellyfish-stings (accessed](https://www.healthdirect.gov.au/jellyfish-stings%20(accessed) 8.7.25)

Newman-Martin, G., 2007. Manual of envenomation and poisoning: Australian fauna and flora. Defence Publishing Service.

Nomura, J.T., Sato, R.L., Ahern, R.M., Snow, J.L., Kuwaye, T.T., Yamamoto, L.G., 2002. A randomized paired comparison trial of cutaneous treatments for acute jellyfish (*Carybdea alata*) stings. Am. J. Emerg. Med. 20, 624–626. 10.1053/ajem.2002.35710

Ohtaki, N., Oka, K., Sugimoto, A., Akizawa, T., Yasuhara, T., Azuma, H., 1990. Cutaneous reactions caused by experimental exposure to jellyfish, *Carybdea rastonii*. J. Dermatol. 17, 108–114. 10.1111/j.1346-8138.1990.tb03716.x

Péron, F., Lesueur, C.A., 1809. Des caractères génériques et spécifiques de toutes les espèces de méduses connues jusqu’ à ce jour. annales du muséum d’ histoire naturelle 18, 325–366.

Queensland Poisons Information Centre, 2024. Sea creature bites and stings, [WWW Document]. URL <https://www.poisonsinfo.health.qld.gov.au/bites-and-stings/sea-creature-bites-and-stings> (accessed 8.7.25)

Reynaud, M., 1830. La carybdée (méduse) ailée”. in: Lesson. R.P., centurie zoologique, ou choix d’animaux rares, nouveaux ou imparfaitement connus. Chéz F.G. Levrault. 95.

Southcott, 1963. State Library of South Australia: PRG 233/9.10 (L213-214), Dr. R. V. Southcott, Letters from Dr. R. V. Southcott to D. J. Lee.

Southcott, R., 1967. Revision of some Carybdeidae (Scyphozoa: Cubomedusae) including a description of the jellyfish responsible for the “Irukandji syndrome.” Aust. J. Zool. 15, 651. 10.1071/ZO9670651

St John Ambulance, 2022. First aid fact sheet: Bites and stings quick guide, [WWW Document].https://stjohn.org.au/assets/uploads/fact%20sheets/english/Fact%20sheets_bites%20and%20stings.pdf (accessed 8.7.25 )

Surf Life Saving Australia, 2020. Coastal safety fact sheet - marine stingers – jimble.

Surf Life Saving Queensland, 2025. Marine stingers – jimble, [WWW Document]. URL <https://lifesaving.com.au/safety-info/marine-stingers> (accessed 11.8.25)

Surf Life Saving South Australia, 2025. Safety fact sheet- Jimble (*Carybdea*), [WWW Document]. URL <https://www.surflifesavingsa.com.au/safety-fact-sheets> (accessed 22.5.25)

Thomas, C.S., Scott, S.A., Galanis D. J., Goto R. S., 2001a. Box jellyfish (*Carybdea alata*) in Waikiki. The analgesic effect of sting-aid, Adolph’s meat tenderizer and fresh water on their stings: a double-blinded, randomized, placebo-controlled clinical trial. Hawaii Med. J. 60.PMID:11573317

Thomas, C.S., Scott, S.A., Galanis, D.J., Goto, R.S., 2001b. Box jellyfish (*Carybdea alata*) in Waikiki: Their influx cycle plus the analgesic effect of hot and cold packs on their stings to swimmers at the beach: A randomized, placebo-controlled, clinical trial. Hawaii Med. J. 101. PMID:11383098

Tibballs, J., 2006. Australian venomous jellyfish, envenomation syndromes, toxins and therapy. Toxicon 48, 830–859. 10.1016/j.toxicon.2006.07.020

Werner, B., 1973. New investigations on systematics and evolution of the class Scyphozoa and the Phylum Cnidaria, Publications of the Seto Marine Biological Laboratory, 20, 35-61

Williamson, J.A., Fenner, P.J., Burnett, J.W., 1996. Venomous and poisonous marine animals: A medical and biological handbook. University of New South Wales Press; Surf Life Saving Queensland.
